# Equity for excellence in academic institutions: a manifesto for change

**DOI:** 10.12688/wellcomeopenres.16861.1

**Published:** 2021-06-07

**Authors:** Lauren Wedekind, Andrés Noé, Jolynne Mokaya, Cynthia Tamandjou, Melissa Kapulu, Andrea Ruecker, Evelyn Kestelyn, Machilu Zimba, Elham Khatamzas, Alice Chi Eziefula, Claire L. Mackintosh, Roger Nascimento, Proochista Ariana, Denise Best, Edward Gibbs, Susanna Dunachie, Gina Hadley, Hannah Ravenswood, Bernadette Young, Charles Kamau, Kevin Marsh, Helen McShane, Rob Hale, Elena McPhilbin, Pavel V. Ovseiko, Oxford Equity in Academia consortium, Rebecca Surender, Claire Worland, Lisa J. White, Philippa C. Matthews

**Affiliations:** 1 Nuffield Department of Medicine, University of Oxford, Oxford, UK; 2 National Institute of Diabetes and Digestive and Kidney Diseases, National Institutes of Health, Phoenix, Arizona, USA; 3 Division of Health Economics, School of Public Health and Family Medicine, Faculty of Health Sciences, University of Cape Town, Cape Town, South Africa; 4 KEMRI Wellcome Trust, Kilifi, Kenya; 5 Mahidol-Oxford Tropical Medicine Research Unit, Faculty of Tropical Medicine, Mahidol University, Bangkok, Thailand; 6 Oxford University Clinical Research Unit (OUCRU), Hanoi, Vietnam; 7 Equality and Diversity Unit, University of Oxford, Oxford, UK; 8 Department of Medicine III, University Hospital, LMU, Munich, Germany; 9 Regional Infectious Diseases Unit, Western General Hospital, Edinburgh, UK; 10 University of Sussex, Falmer, Brighton, UK; 11 International Health and Tropical Medicine, Centre for Tropical Medicine and Global Health, Medawar Building, Nuffield Department of Medicine, University of Oxford, Oxford, UK; 12 Oxford University Clinical Academic Graduate School (OUCAGS), John Radcliffe Hospital, Oxford, UK; 13 Department of Tropical Medicine, Nuffield Department of Medicine, University of Oxford, Oxford, UK; 14 Department of Infectious Diseases and Microbiology, Oxford University Hospitals NHS Trust, John Radcliffe Hospital, Oxford, UK; 15 Department of Medicine, Oxford University Hospitals NHS Trust, John Radcliffe Hospital, Oxford, UK; 16 Harris Manchester College, Oxford, UK; 17 Africa Oxford Initiative, Medawar Building, University of Oxford, Oxford, UK; 18 NIHR BRC, Oxford University Hospitals NHS Trust, John Radcliffe Hospital, Oxford, UK; 19 MaynardLeigh Associates, London, UK; 20 Radcliffe Department of Medicine, John Radcliffe Hospital, Oxford, UK; 21 Department of Social Policy, Social Sciences Division, University of Oxford, Oxford, UK; 22 Big Data Institute, University of Oxford, Oxford, UK

**Keywords:** Equity, diversity, inclusion, academia, publishing, retention, gender, race, Athena SWAN, Black Lives Matter, Race Equality Charter, STEMM

## Abstract

Higher academic institutions in the UK need to drive improvements in equity, diversity, and inclusion (EDI) through sustainable practical interventions. A broad view of inclusivity is based on an intersectional approach that considers race, geographical location, caring responsibilities, disability, neurodiversity, religion, and LGBTQIA+ identities. We describe the establishment of a diverse stakeholder group to develop practical grass-roots recommendations through which improvements can be advanced. We have developed a manifesto for change, comprising six domains through which academic institutions can drive progress through setting short, medium, and long-term priorities. Interventions will yield rewards in recruitment and retention of a diverse talent pool, leading to enhanced impact and output.

## Disclaimer

The views expressed in this article are those of the authors. Publication in Wellcome Open Research does not imply endorsement by Wellcome.

## Introduction

Academic institutions have a moral imperative to provide an environment in which every individual has the rights and opportunities to participate and develop to their greatest potential ^1^. Beyond the case for justice, promoting equity, diversity, and inclusion (EDI), and prioritising accessibility, also stimulates collaboration, output, innovation, and impact ^2, 3^. A positive spillover of skills and knowledge results from sharing different perspectives ^4^, and cultural and cognitive diversity represent different knowledge, external linkages, and social intelligence ^5^. Individuals who feel valued and psychologically safe within their working environments contribute more fully ^6^, benefiting academic institutions and their partners in healthcare, education, industry, the charity sector, and public engagement, while a diverse workforce can contribute to better governance and ethics and generate output that better represents society ^7, 8^.

The private sector has increasingly invested in the strong ‘business case’ for EDI ^9, 10^, perhaps because there is a demand for diversity from clients, but also as a result of realising that to benefit from the full talent pool, organisations must recruit and promote members from diverse backgrounds ^11^. Conversely, individuals who feel under-valued and under-recognised are more likely to underperform and/or leave their roles ^12^. Teams that prioritise EDI build resilience, strengthening their abilities to resolve conflict, address bias, and cope with stress ^13, 14^.

The measures of excellence for academic institutions are beginning to shift from outdated metrics (e.g., weighted towards academic publication) ^15^ towards more holistic measures of impact ^16^. Academic funders increasingly demand delivery of impact through collaborative networks, and expect engagement with the public, research participants, and the private sector ^17^. Likewise, research funders have introduced specific EDI mandates ^18, 19^, while student and staff bodies are demanding reform.

By simply maintaining the *status quo*, institutions neglecting EDI as a priority risk being overtaken by more diverse competitors, suffering legal, financial, and reputational consequences, losing resources and collaborations, and further minoritising historically excluded groups ^12^. Sticking to a ‘business as usual’ approach for EDI is insufficient. The Athena SWAN Charter, established in the UK in 2005, has delivered some progress in this domain by providing a structured framework to drive improvements in gender equity through action plans, benchmarking, and funding incentives ^20, 21^. Although it has been criticised for its ‘tick-box’ approach, the Charter has demonstrably driven awareness and promoted investment, underpinned training, and influenced practical changes. More recently, Athena SWAN has undergone review, extending its remit beyond gender to other identities ^22^, and recognising the need for intersectional approaches ^23^ alongside the establishment of other frameworks such as the Race Equality Charter (REC) ^24^ and the Stonewall Workplace Equality Index ^25^.

There is now a need to increase the pace of change, while adapting to major global health, political, and climate challenges ^26^. We convened a consultative ‘Equity in Academia’ symposium in September 2019 ^27^, led through the Nuffield Department of Medicine at the University of Oxford and its international partners, aiming to position EDI as a cornerstone of university activity, making practical changes within faculty, research, communication, leadership, policy, planning, and resource allocation. Our working party included clinicians, academics, and faculty staff at different career stages, including diverse geographical representation facilitated through the Oxford Tropical Network, working in consultation with academic publishers, leaders from our institutional Equity and Diversity networks, and experts in teaching, communication, and human resources. We collected qualitative data before, during, and after the event (available on-line) ^28^.

Since our meeting was convened, the global academic and clinical communities have undergone dramatic changes. The COVID-19 pandemic is associated with wide-ranging influences as a result of population lock-down and repurposing of resources, with the potential for disproportionate negative impact on already minoritised groups ^29, 30^. Mandates for equity, including Athena SWAN targets, have been set aside. Special efforts will be required to monitor and mitigate wide-reaching effects of this global emergency, and to ensure that progress made towards EDI goals is not eroded. The Black Lives Matter movement ^31^ has raised enhanced awareness of ingrained societal inequities based on race, present long before COVID-19, but intersecting with the impact of the pandemic on minoritised and disadvantaged groups ^32, 33^.

In the body of this article, we summarise practical recommendations, initially developed as an output from our meeting, but subsequently shaped further through consultation with wider EDI stakeholder groups and based on adaptation to the changing landscape.

## Recommendations for change

**Figure 1.**
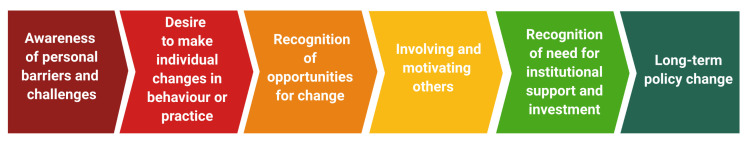
Evolution of events in driving institutional change for improvements in equity and diversity. Summary based on feedback and reflection of participants before, during, and after the Equity in Academia event. Personal contributions and activism are crucial in designing new approaches to promote EDI, but investment from leadership and dialogue with stakeholders at the institutional level are needed to enforce sustainable policy changes that ultimately become embedded in policy.

We present an overarching manifesto comprising six key domains for change, leading to practical recommendations wherever possible. We have considered the timelines over which we anticipate change might be implemented and predicted the resource impact of different targets ( Table 1), to help set and deliver focused goals. The aim is to underpin and support strategic change in ways that can be implemented in practice ( Figure 1), can be benchmarked, are transparent and become a sustainable investment over time. Our list does not propose to be exhaustive, and individual recommendations should not be addressed in isolation, but rather as part of a developing network of initiatives, with an understanding that particular recommendations need to be adapted or prioritised according to local needs and resources, and tailored to make them context-specific.

**Table 1.** Recommendations for supporting initiatives for equity, diversity, and inclusion (EDI) at academic institutions. We divide our recommendations into six broad domains. Each target is classified according to the likely speed and cost of implementation, to support the development of realistic priorities and timelines. However, we recognise that these attributes vary by time and place, and therefore recommend context-specific evaluation, and frequent reappraisal of goals, backed by relevant local data.

Recommendation	Speed	Cost
Domain one: Improve representation		
(i) Invest in visible representation from high level leadership	short	Medium
(ii) Improve diversity in university leadership and management roles	Medium	Low
(iii) Improve inclusion of historically excluded and under-represented groups across all roles	Medium	Medium
(iv) Develop a cohesive organisational structure for equity	Medium	Medium
Domain two: Commit resources		
(i) Develop and maintain networks for best practice	Long	High
(ii) Commit to equitable funding	short	Medium
(iii) Ring-fence funding to work towards targets set by the Race Equality Charter	Medium	Medium
(iv) Collect and analyse data	Medium	High
Domain three: Invest in rewards and recognition		
(i) Re-focus central institutional reward schemes	Medium	Medium
(ii) Offer development opportunities for early career researchers	short	Medium
(iii) Create new career opportunities in EDI	Medium	High
Domain four: Tackle bullying and harassment		
(i) Tackle challenges and complaints through a consistent framework	short	Medium
(ii) Collect and act on leavers’ data	short	Medium
Domain five: Build opportunities		
(i) Drive improvements in EDI in academic publishing	Long	High
(ii) Make the workplace accessible to all	Medium	Medium
(iii) Invest in skills, training, and support for EDI initiatives	Medium	Medium
(iv) Recalibrate entry requirements	short	Low
Domain six: Support equity through policy		
(i) Apply legislation, policy, and expectations universally	Medium	High
(ii) Move from policy into practice by setting specific goals and targets	Medium	Medium

### Domain one: Improve representation

*(i) Invest in visible representation from high level leadership*

Visible leadership in EDI is essential to demonstrate a commitment to delivering practical policy change, and universities are increasingly creating dedicated senior EDI positions. Many of our recommendations require a level of power, influence, or authority within the structure of an institution, requiring representative leadership to deliver and sustain improvements. In addition to designated roles that incorporate an EDI remit, all individuals in leadership positions should be required and incentivised to commit time and resources to improving EDI. However, careful management of targets is required to ensure that outputs cannot be fudged or linked to perverse incentives. Grass-roots change can only be sustained if there is evidence of this personal, practical, and economic investment ^34^. 

*(ii) Improve diversity in leadership and management roles*

Increased effort is required to ensure that panels, committees, and governing boards are as diverse as possible, beyond gender identities alone. To develop an organisational ethos that is intersectional, and to avoid segregation of groups representing different sectors and backgrounds, this diversity must be partnered with an approach that champions inclusion and integration, also offering opportunities to individuals at junior career levels. Contributions to activities that promote EDI should be recognised, rewarded and valued, and responsibilities must be shared to ensure fair distribution of the workload, to avoid a minority of individuals bearing an undue weight of responsibility, especially if these are people who do not represent the existing norms of an institution ^35^. Members of well represented groups need to step up on advocacy and allyship ^36^, not just leaving the agenda to be promoted by members of minoritised groups.

*(iii) Improve inclusion of historically excluded and under-represented groups across all roles*

Efforts are required to improve inclusion and intersectionality, with respect to (non-exhaustively) gender, disability, mental or physical illness, neurodiversity, ethnicity, geographical location, and LGBTQIA+ identities. The global South and low/middle income countries (LMIC) remain under-represented among staff and students, and in the academic culture, curriculum, resources and reading lists; research contributions from LMIC settings are often unfairly weighted with disproportionate credit to academic partners in resource-rich environments ^37^. Expertise in advertising roles and opportunities is needed to ensure visibility to a wide audience. Blind recruitment may have a role in reducing the impact of (conscious or unconscious) bias in recruitment panels, while mandating diverse short-listing and interview panels would improve equity and inclusivity in decision making.

Institutions can designate dedicated international scholarships and visiting fellowships to enhance mutually beneficial collaboration, with administrative support to provide assistance with travel arrangements. These opportunities can underpin career development and collaboration, alongside inclusion in committees, panels and steering groups, including roles in development and decolonisation of academic curricula, and providing access for minoritised groups to career development and leadership opportunities. Specific de-colonization of the academic sector is particularly central to EDI interventions in countries with traumatic colonial histories.

*(iv) Develop a cohesive organisational structure for equity*

Many individual efforts are currently made to improve EDI, but often occur in poorly connected silos. A minimum of one annual meeting at university-wide level is suggested, including representation from international partners, as an opportunity for networking, training, sharing challenges and experiences, convening focus groups, building consensus, and generating practical recommendations. This would also be a valuable opportunity to collect data, invite external speakers and network with other organisations. Such events must be accessible in format, timing, and venue.

### Domain two: Commit resources

*(i) Develop and maintain networks for best practice*

Investment is required to establish an institution-wide network that builds and maintains personal connections, hosts case studies, catalogues useful references and resources, promotes funding opportunities, and contributes to the organisation of EDI meetings. While we work in a financially challenging climate, budget should be ring-fenced to support and sustain activities, with consideration of how funding is most inclusively deployed and a recognition that investment in this arena leads to longer-term dividends in productivity. Specific incentivisation may be needed within departments to ensure short-term cuts do not curtail progress. Expertise in EDI leadership can thus be grown, alongside the coordination of departmental platforms and resources to support and sustain activity. Investment in new initiatives can improve EDI at an organisational level (local examples are ‘Foundation Oxford’ and ‘Opportunity Oxford’) ^38^.

*(ii) Commit to equitable funding*

At present, university data show disparities in gender and ethnic representation and pay. Transparent audit and data are required to underpin policies and implement change ^39^.

Individuals who take on roles to promote EDI are often already disadvantaged as members of minoritised groups themselves, and typically do so in their own time without compensation, and at a detriment to other personal and professional responsibilities; this phenomenon has been coined the ‘minority tax’ ^40^. University funds are needed to support flexible work arrangements, administrative and research staff, IT, travel, and carers’ and hardship funds. EDI activity needs to be formalised in job plans, including participation on committees, or working groups. Academic units should generate written annual commitments to EDI activity, with audit and benchmarking to ensure that these commitments are delivered.

*(iii) Ring-fence funding to work towards targets set by the Race Equality Charter*

Goals and targets set by the Athena SWAN Charter for gender equity have taken priority, due to financial incentives for delivery. However, a wider commitment is now required to promote EDI in domains beyond gender. Practical institutional responses to issues raised by the Black Lives Matter campaign are urgently required; for example, Oxford University established the Race Equality Task Force in November 2020, providing a unified approach through which to tackle racial discrimination and drive practical advances in the REC action plan. An active anti-racist stance requires culture change, consistent investment of expertise and resources, and regular scrutiny through benchmarking against targets ^26^.

*(iv) Collect and analyse data*

Data collection and analysis is essential to identify diversity gaps and focus resources appropriately to tackle the areas of greatest need ^41, 42^, which may vary between organisations and even between departments in a single institution. Undertaking this in a meaningful way requires the allocation of time, resources, and skills. Having identified and agreed specific EDI targets, progress should be tracked through repeat data collection and audit, to affirm positive outcomes and to identify areas where further effort is required. Regular public reporting of such data is good practice and an incentive to build on progress ^43^. A challenge for data analysis is the trade-off between the value of disaggregated data in order to develop a high-resolution picture, versus the risk of raising privacy issues. Using external agencies to analyse data and make recommendations is a potential way to address this concern, removing the risk of specific individuals being identified.

### Domain three: Invest in rewards and recognition

*(i) Re-focus central institutional reward schemes*

Many universities offer merit awards and have programmes for recognition of distinction, typically focusing on publication and funding as metrics. However, the impact that can be delivered through teaching, leadership, and EDI roles should be recognised and rewarded as equally important domains. Although equity awards are currently offered, such opportunities need to be expanded, advertised, and championed, with consideration of appropriate metrics or key performance indicators. New ‘ambassador’ roles could support individuals who deliver impact in improving EDI. Selection panels for recognition of distinction must themselves represent and champion diversity.

*(ii) Offer development opportunities for early career researchers*

Existing ‘honorary visiting research fellowship’ and ‘visiting professorship’ schemes recognise mid/senior career stages, enhancing geographical diversity, and providing career opportunities. We propose the addition of a similar title for early-career researchers; for example, ‘junior visiting research fellowships’ with college or departmental affiliations could be introduced, together with a parallel series of awards that support projects with a specific EDI focus.

*(iii) Create new career opportunities in EDI*

Academic institutions should consider the creation of more specific EDI posts, as well as creating flexibility in compensated positions to formally protect time for EDI work in parallel with other roles, with these opportunities advertised alongside other posts. This would require the provision of extra resources to allow appointed applicants to undertake a formal ‘equity brief’.

### Domain four: Tackle bullying and harassment

*(i) Tackle challenges and complaints through a consistent framework*

The individual complaints procedure is well documented in most institutions, with formal frameworks through which concerns are addressed. Unfortunately, it remains the case that culture and job security challenges often inhibit people from coming forward. One way to protect individuals is to collate any complaints which may indicate potentially deeper systemic problems to be strategically addressed at an institutional level, rather than as a series of unrelated issues. Responses to complaints should be planned directly with stakeholders (including issues that may be raised outside the formal complaints process). Serious concerns should be handled at a departmental or organisational level to liberate the individual complainant from the onus of personal responsibility or fears of individual repercussions. Dedicated mechanisms can be implemented to collate concerns that should be dealt with collectively, such as racism or harassment. Complaints should be dealt with efficiently to demonstrate a ‘zero tolerance’ policy, strict adherence to the law, and reflecting institutional and funders’ policies ^39^.

*(ii) Collect and act on leavers’ data*

Improved transparency and enhanced sensitivity are required in the collection and analysis of data at ‘exit interviews’ to identify hostile environments, and to spotlight micro- and macro-aggression that cause academic staff and faculty members to leave their positions ^44^. This opportunity for learning is a well recognised process but in practice is not consistently undertaken, or acted upon. More efforts to understand the optimum ways to collect and learn from such data are needed, and to determine the value of the process in order to justify ongoing investment.

### Domain five: Build opportunities

*(i) Drive improvements in EDI in academic publishing*

More data are needed to understand, highlight, and tackle equity gaps in academic publishing ^45, 46^, including editorial membership and peer review ^43, 47^. By investing in acquiring, analysing, and publishing its own data regarding EDI in authorship ^15^, academic institutes can inform local improvements, and leverage publishers to demonstrate equity and diversity among authors, reviewers, and editors. Universities should lobby for provision of training in publishing and peer review, making this relevant and accessible to researchers in LMICs with costs shared by publishers. Academic trainees, faculty, administrators, and support staff need opportunities and incentives to collaborate, research and publish on EDI topics to share experiences and influence institutional improvements. Action is required to ensure that subjects and participants of research are represented equitably (e.g., advocating for improved diversity of representation of study subjects by age/sex/ethnicity) ^48^.

*(ii) Make the workplace accessible to all*

Greater resource and staff allocation are required to modify and improve workplace, living and conference environments to cater to diverse needs of the workforce, including such considerations as step-free access, accessible public transport or car parking, hearing loops, sign language interpretation, and captioning. Childcare, parental leave, and flexible working hours must be championed to support all those with caring responsibilities, including - but not exclusive to - working parents and carers.

*(iii) Invest in skills, training, and support for EDI initiatives*

Greater commitment is required to provide programmes that build and nurture transferable skills, such as communication, negotiation, leadership, advocacy, self-confidence, and empathy, breaking down stereotypes, and (implicit and explicit) bias that may inhibit minoritised communities. Resource is needed to provide high quality training that is available both on-line and face-to-face, and should be mandated for those in teaching, mentorship, leadership, or recruitment roles. Prudent investment recognises the merits of intersectional approaches and draws on successful initiatives in other sectors (e.g., industry, healthcare, military, and the media). Mentorship and sponsorship schemes that are supported through university investment need to be flexible and adaptive to the needs, interests, and challenges of the mentee, and can be used to develop and maintain connections between teams and departments. A diverse view of relevant and valuable skills is needed, to broaden the nature of training that is provided and to dismantle traditional views of leadership which perpetuate the status quo.

*(iv) Recalibrate entry requirements*

Admissions to posts across all institutional levels (e.g., internships, studentships, and professorships) should be holistically reviewed, accounting for barriers that the applicant may have had to overcome ^49^, adjusting pro-rata for career breaks, and considering the potential that they show beyond publications, impact factors, grade-point averages and standardised test scores.

### Domain six: Support equity through policy

*(i) Apply legislation, policy, and expectations universally*

Universities should uphold and publicise universal mandates to deliver on EDI goals, such that expectations are explicitly shared with affiliated institutions, collaborators, and partners in other domains, such as industry and the charitable sector. Infrastructure and resources are needed to support aspirations of teams who can exemplify excellence and deliver output that sustainably improves institutional EDI, such as actively prioritising targets set by the Racial Equality Charter.

*(ii) Move from policy into practice by setting specific goals and targets*

Following review and agreement within the appropriate domain, mandates for changes involving domains one to five above should be enshrined in policy, with active monitoring and sanctions applicable if not delivered.

## Discussion

Based on output from an international multi-disciplinary symposium, wide consultation with relevant stakeholders and experts, and review of a range of published sources, we have developed a manifesto of recommendations for enhancing EDI at academic institutions. While there is a diverse literature in this field, it remains the case that consistent, evidence-driven approaches to enhancing EDI have not been widely adopted across academia. We have therefore set out to unify expertise and experience, in order to collate recommendations into an accessible format to help inform EDI interventions in complex university environments, with the aim of providing leverage for meaningful and sustainable change.

Beyond the moral imperative, prioritising EDI through the ‘innovation economy’ can deliver returns on investment, through diversifying research and teaching, accessing a larger pool of talent, and increasing the likelihood of winning competitive funding. The case for reputation recognises that there are inherent risks for institutions that fail to keep pace. While the reasons for prioritising EDI are well recognised, there is a gap between ‘knowing’ and ‘doing’ within organisations ^9^, and it has been too easy to adopt a passive approach rather than actively challenging inequities, recently highlighted by calls for anti-racism in science ^43^. To close these gaps, resources must be sustainably invested in an organisation-wide EDI agenda with high-level institutional support. We recognise that different organisations, institutions, and disciplines have their own complex systems and cultures, which need to be accounted for when planning change, such that interventions have the maximum chance of success in that specific context. An example is ‘women in chemistry’ ^50^, through which challenges in a particular arena are both highlighted and addressed.

Universal EDI policies and standards should be developed upon consultation with wide stakeholder groups, and transparently scrutinised, so that expectations are consistent across departments, divisions, and institutions, irrespective of geography. Reliance on self-selected volunteers (typically from minoritised groups) to drive advocacy, collect data, manage projects, and implement changes can no longer be accepted ^51^.

EDI policy changes and enforcement are complex and far-reaching. Academic institutions work with limited resources that must be shared across domains, with potentially competing risks and benefits. Some interventions and policies designed to support EDI carry the risks of unintended consequences; without stakeholder engagement and a receptive environment, a ‘backlash’ effect can paradoxically cause the most harm to the groups that a policy set out to support. Likewise, we must recognise and avoid the ‘deficit model’ where solutions are aimed at changing the individual instead of addressing a problem in the system, culture, or institution ^24^.

Data collection is essential to focus activities to promote EDI, but requires expertise and resources, and can be difficult in the complex multi-dimensional space of a university, in which the impact of an intervention may not be easy to measure. Institutions must avoid imposing an additional layer of pressure and demands on minoritised and historically excluded groups, for whom opportunities are sometimes offered alongside an unrealistic expectation that their involvement will inherently and immediately deliver improvements. True commitment to EDI must be managed with shared responsibility and realistic timeline expectations, being mindful of potential challenges and considering how to measure success of a programme or intervention.

The academic model in which individual achievement is recognised and rewarded only through metrics of publication and grant income is slowly being diversified. However, further incentives for change will be crucial to dismantle existing this outdated approach to appraisal, for example recognising distinction in fields that include leadership and citizenship, teaching, collaborations, EDI commitments and public engagement.

Complete strategic change is required to put EDI at the heart of policy for higher education institutions. The COVID pandemic has taught us that academic institutions can be agile in making changes at speed when deemed essential to sustain activity and productivity; if we can capitalise on the same innovation and investment for EDI interventions, then there is potential for huge progress ^52^. Interventions need to be resourced, developed, and delivered with consideration of positive impact, while offsetting potential risks and costs, scrutinised through benchmarking and feedback, and delivered with transparency and sustainability. 

## Data availability

### Underlying data

Figshare: Equity in Academia: Supporting Material collected through a workshop at Oxford University (September 2019). https://doi.org/10.6084/m9.figshare.13849964.v2 ^28^.

The project contains the following underlying data:

- EIA supplementary data.pdf (qualitative information collected from meeting delegates, before, during and after the workshop)
- Equity in Academia.jpg 1–14 (visual representations of the day's events, summarising themes and discussions. Artwork produced by Alex Hughes at Drawnalism ( https://drawnalism.com/), posted with permission.)

### Extended data

Figshare: Equity in Academia: Supporting Material collected through a workshop at Oxford University (September 2019). https://doi.org/10.6084/m9.figshare.13849964.v2 ^28^.

This project contains the following extended data:

• s16861-V1-2-Suppl_Table_1_210421_Equity_in_Academia_-_consortium_authorship_list.xlx (Consortium author list and affiliations). All the individuals named in the consortium have agreed to have their names and affiliations published and available in a publicly accessible repository.

Data are available under the terms of the Creative Commons Attribution 4.0 International license (CC-BY 4.0).
