# Supplementary material for: Equity for excellence in academic institutions: a manifesto for change
Source: Wellcome Open Res. 2021 Jun 7;6:142. [Version 1] doi: 10.12688/wellcomeopenres.16861.1 (PMC8343400; doi:10.12688/wellcomeopenres.16861.1)
Supplement: Peer review history

# Peer review: Reviewer response for version 1

**DOI:** 10.21956/wellcomeopenres.18599.r44872

**Published:** 2021-08-05

**Authors:** Rachel Roper

**Affiliations:** 1 Department of Microbiology and Immunology, Brody School of Medicine, East Carolina University, Greenville, NC, USA

Equity for excellence in academic institutions: a manifesto for change is a valuable article that proposes many useful steps to improve equity, diversity, and inclusion (EDI, or DEI in some geographic regions, e.g. the US). The authors do an excellent job in describing or reminding readers of the many and various ways that diversity is important and beneficial to everyone.  

 Figure 1 shows an image of required steps for improvements. I particularly like step 1, the “Awareness of personal barriers and challenges”. Those who do not believe there is any problem with equity and diversity often suggest that women, minority, or other historically underrepresented groups (URGs) do not have the aptitude or desire to work in such professions, do not work hard enough, or perhaps want to be homemakers rather than be in the workforce. This is why it is crucial to publicize and educate faculty on the many well-designed studies that demonstrate statistically significant gender and racial bias in academia today (reviewed in reference 3, Roper RL: Does Gender Bias Still Affect Women in Science? *Microbiol Mol Biol Rev.* 2019; 83(3): e00018–19, and Eaton *et al.* How Gender and Race Stereotypes Impact the Advancement of Scholars in STEM: Professors’ Biased Evaluations of Physics and Biology Post-Doctoral Candidates, June 2019 *Sex Roles.*) ^1^ These studies show that it is the faculty and administrators today who are controlling the success and careers of others, and it is we who can effect change.

             The authors make an excellent recommendation to have a minimum of one annual meeting at university-wide level to address EDI efforts, encourage crosstalk between different units and harmonize efforts. This will have the additional benefit of raising awareness and reminding all of campus about EDI. At such a meeting, updates could be given on the diversity data of the institution and comparisons made to national or international norms of faculty and student composition. For example, in the USA, we have the National Faculty Distribution Survey that Oklahoma State University administers every two years covering approximately 80 U.S. institutions and 100,000 faculty, giving data on gender and minority faculty. Universities can compare their departments to national averages to see if there are particular departments that are far below the norms and which may need particular attention from administration. The authors correctly point out the difficulty of presenting data on small sets such as departments, where privacy issues can be a concern. In the US for instance, a person’s gender is protected data, so often only data sets larger than 5 can be publicly discussed or presented. Larger aggregates of data can be presented. However, acquiring the data is a fundamental first step. We must know where we are in order to measure progress.

             The article underscores that those who promote EDI “are often already disadvantaged as members of minoritised groups themselves, and typically do so in their own time without compensation, and at a detriment to other personal and professional responsibilities; this phenomenon has been coined the ‘minority tax’”. Since this work is much needed, it is an imperative for universities to officially recognize and reward such time and effort. Many faculty are judged based on three categories; research (publication and grants), teaching and service. It may be wise to add a fourth official category to impress its importance since it seems to be often overlooked and undervalued. Research indicates that faculty may be the most effective at delivering EDI training to faculty since they share the same community, culture and language (Smith, *et al. *2015 Now Hiring! Empirically Testing a Three-Step Intervention to Increase Faculty Gender Diversity in STEM *BioScience*, and Devine *et al. *2017. A Gender Bias Habit-Breaking Intervention Led to Increased Hiring of Female Faculty in STEMM Departments. *J Exp Soc Psychol*.), ^2^^,^^3^ so recognizing and rewarding faculty for this work is crucial.

 The authors suggest that blind (anonymous) recruitment or evaluation may have a role to avoid bias in selection. There is support for this in a recent study on applications for time on the Hubble Space telescope. When applications were made anonymous the success rate for women increased dramatically and for the first time was higher than for men (Dual-anonymization Yields Promising Results for Reducing Gender Bias: A Naturalistic Field Experiment of Applications for *Hubble Space Telescope* Time, Johnson and Kirk 2020 *. The Astronomical Society of the Pacific*). ^4^ However, anonymizing applications cannot make up for poverty, poor early circumstances in educational opportunities, or accumulated bias over the applicant’s lifetime. For example, in the US, American schools have become segregated by race again, and predominantly white school districts have collectively $23 billion more per year indicating that racial/ethnic minority groups receive fewer educational opportunities in lower quality schools ( https://www.npr.org/sections/live-updates-protests-for-racial-justice/2020/07/07/888469809/how-funding-model-preserves-racial-segregation-in-public-schools ). This then, in turn, may result in reduced success in college and graduate school. The US National Science Foundation reports that only 8.8 % of science and engineering PhD’s were awarded to minority people in 2015 ( https://www.nsf.gov/statistics/2017/nsf17306/report/who-earns-a-us-doctorate/race-and-ethnicity.cfm). Since having a PhD is normally a prerequisite for a faculty position, this makes hiring a representative proportion of diverse faculty from historically underrepresented groups (URG) a near impossible challenge. Search and admissions committees must evaluate the circumstance of the applicants and not just obvious accomplishments that are much easier for a person with privilege to obtain. Faculty should also encourage individuals from historically underrepresented groups to go to graduate school for advanced degrees, and funding should be available to support them.

 The article points out the need for EDI to be enshrined in policy. There are many ways that this can be accomplished. It can be written into job descriptions for department chairs and graduate student directors, that they have a responsibility to create and ensure an atmosphere supportive of diversity, equity and inclusion. Policies should require equity in space, resources and the amount of start up funds faculty members receive. Universities can institute multiple levels of promotion and/or tenure review so that if a department/unit votes against a person, it can be reviewed by an upper level, perhaps school-wide committee. Since EDI is often simply forgotten in normal department functioning, it can be written into unit/department governance codes and procedures to remind everyone to think about and pay attention to diversity issues in: evaluation of how faculty members treat diverse students, in any analysis of salary increases, for service work in DEI, and the extra mentoring work that minority/historically underrepresented faculty end up doing helping URG students and supporting them to enter what may be historically white and male dominated fields. Raising awareness of bias, reminding faculty of EDI during normal operations, monitoring data, and giving credit for EDI work will have important benefits in improving EDI at universities.

Does the article adequately reference differing views and opinions?

Yes

Are all factual statements correct, and are statements and arguments made adequately supported by citations?

Yes

Is the Open Letter written in accessible language?

Yes

Where applicable, are recommendations and next steps explained clearly for others to follow?

Yes

Is the rationale for the Open Letter provided in sufficient detail?

Yes

Reviewer Expertise:

Gender bias in STEMM, poxvirology, coronaviruses

I confirm that I have read this submission and believe that I have an appropriate level of expertise to confirm that it is of an acceptable scientific standard.

## References

[1] : How Gender and Race Stereotypes Impact the Advancement of Scholars in STEM: Professors’ Biased Evaluations of Physics and Biology Post-Doctoral Candidates. *Sex Roles* .2020;82(3-4) : 10.1007/s11199-019-01052-w 127-141 10.1007/s11199-019-01052-w

[2] : Now Hiring! Empirically Testing a Three-Step Intervention to Increase Faculty Gender Diversity in STEM. *Bioscience* .2015;65(11) : 10.1093/biosci/biv138 1084-1087 10.1093/biosci/biv138 26955075PMC4777060

[3] : A Gender Bias Habit-Breaking Intervention Led to Increased Hiring of Female Faculty in STEMM Departments. *J Exp Soc Psychol* .2017;73: 10.1016/j.jesp.2017.07.002 211-215 10.1016/j.jesp.2017.07.002 29249837PMC5729935

[4] : Dual-anonymization Yields Promising Results for Reducing Gender Bias: A Naturalistic Field Experiment of Applications forHubble Space Telescope Time. *Publications of the Astronomical Society of the Pacific* .2020;132(1009) : 10.1088/1538-3873/ab6ce0 10.1088/1538-3873/ab6ce0

# Peer review: Reviewer response for version 1

**DOI:** 10.21956/wellcomeopenres.18599.r44330

**Published:** 2021-07-05

**Authors:** Juan Sanabria

**Affiliations:** 1 Department of Surgery, Marshall University Joan Edwards School of Medicine, Huntington, WV, USA

Authors present elegantly a manifesto of ideas to increase and maintain diversity and equity in academic and perhaps, non-academic health systems and organizations. 

 Although I agree enterally with the content and aims,  it will require a major shift of culture for each Institution/Organization. In my experience, this is the most difficult change. It takes time, resources, persistence and the buy of the goals by all stakeholders. 

 It would help a timeframe guidance for each goal, the slow-downs and set backs and a practical guidance to overcome the expected fences at different levels.

Does the article adequately reference differing views and opinions?

Yes

Are all factual statements correct, and are statements and arguments made adequately supported by citations?

Yes

Is the Open Letter written in accessible language?

Yes

Where applicable, are recommendations and next steps explained clearly for others to follow?

Yes

Is the rationale for the Open Letter provided in sufficient detail?

Yes

Reviewer Expertise:

Basic and translational cancer biology and metabolism

I confirm that I have read this submission and believe that I have an appropriate level of expertise to confirm that it is of an acceptable scientific standard.
